# The People’s Dental Journal

**Published:** 1863-08

**Authors:** 


THE PEOPLE'S DENTAL JOURNAL. Edited by W. W. ALLPORT, D.D.S., and S. T. CREIGHTON. Quarterly; price 50 cents per annum

The July number of this journal is before us, filled with useful matter for all who are interested in the proper care and preservation of the teeth; and this should include the entire community.

